# Duration of androgen deprivation therapy with postoperative radiotherapy for prostate cancer: a comparison of long-course versus short-course androgen deprivation therapy in the RADICALS-HD randomised trial

**DOI:** 10.1016/S0140-6736(24)00549-X

**Published:** 2024-05-16

**Authors:** Chris C Parker, Howard Kynaston, Adrian D Cook, Noel W Clarke, Charles N Catton, William R Cross, Peter M Petersen, Rajendra A Persad, Cheryl A Pugh, Fred Saad, John Logue, Heather Payne, Lorna C Bower, Chris Brawley, Mary Rauchenberger, Maroie Barkati, David M Bottomley, Klaus Brasso, Hans T Chung, Peter W M Chung, Ruth Conroy, Alison Falconer, Vicky Ford, Chee L Goh, Catherine M Heath, Nicholas D James, Charmaine Kim-Sing, Ravi Kodavatiganti, Shawn C Malone, Stephen L Morris, Abdenour Nabid, Aldrich D Ong, Rakesh Raman, Sree Rodda, Paula Wells, Jane Worlding, Wendy R Parulekar, Mahesh K B Parmar, Matthew R Sydes

**Affiliations:** Royal Marsden NHS Foundation Trust, London, UK; Institute of Cancer Research, London, UK; Division of Cancer and Genetics, Cardiff University Medical School, Cardiff, UK; MRC Clinical Trials Unit at UCL, Institute of Clinical Trials and Methodology, University College London, London, UK; Department of Urology, The Christie NHS Foundation Trust, Manchester, UK; Division of Cancer Sciences, University of Manchester, Manchester, UK; Department of Urology, Salford Royal Hospital, Salford, UK; Princess Margaret Cancer Centre, Toronto, ON, Canada; Department of Urology, St James’s University Hospital, Leeds, UK; Department of Oncology, Rigshospitalet, University of Copenhagen, Copenhagen, Denmark; Department of Urology, Bristol Urological Institute, Bristol, UK; MRC Clinical Trials Unit at UCL, Institute of Clinical Trials and Methodology, University College London, London, UK; Department of Urology, Centre Hospitalier de l’Université de Montréal, Montréal, QC, Canada; Department of Urology, The Christie NHS Foundation Trust, Manchester, UK; The Prostate Centre, London, UK; Royal Marsden NHS Foundation Trust, London, UK; Institute of Cancer Research, London, UK; Guy’s and St Thomas’ NHS Foundation Trust, London, UK; MRC Clinical Trials Unit at UCL, Institute of Clinical Trials and Methodology, University College London, London, UK; MRC Clinical Trials Unit at UCL, Institute of Clinical Trials and Methodology, University College London, London, UK; Department of Radiation Oncology, Centre Hospitalier de l’Université de Montréal, Montréal, QC, Canada; Department of Clinical Oncology, St James’s University Hospital, Leeds, UK; Department of Urology, Copenhagen Prostate Cancer Center, Rigshospitalet, Copenhagen, Denmark; Department of Radiation Oncology, Sunnybrook Odette Cancer Centre, Toronto, ON, Canada; Department of Radiation Oncology, University of Toronto, Toronto, ON, Canada; Princess Margaret Cancer Centre, Toronto, ON, Canada; Department of Radiation Oncology, University of Toronto, Toronto, ON, Canada; Department of Clinical Oncology, The Christie NHS Foundation Trust, Manchester, UK; Radiotherapy Department, Charing Cross Hospital, London, UK; Royal Devon and Exeter University NHS Foundation Trust, Exeter, UK; Royal Surrey County Hospital, Guildford, UK; Department of Clinical Oncology, University Hospital Southampton NHS Foundation Trust, Southampton, UK; Royal Marsden NHS Foundation Trust, London, UK; Institute of Cancer Research, London, UK; Department of Radiation Oncology, BC Cancer—Vancouver, Vancouver, BC, Canada; Glan Clwyd Hospital, Betsi Cadwaladr University Health Board, Bangor, UK; Ottawa Hospital, University of Ottawa, Ottawa, ON, Canada; Guy’s and St Thomas’ NHS Foundation Trust, London, UK; Service de Radio-Oncologie, Centre Hospitalier Universitaire de Sherbrooke, Sherbrooke, QC, Canada; Max Rady Faculty of Health Sciences, University of Manitoba, Winnipeg, MB, Canada; Kent Oncology Centre, Kent and Canterbury Hospital, Canterbury, UK; Bradford Teaching Hospitals, Bradford, UK; Barts Cancer Centre, St Bartholomew’s Hospital, London, UK; University Hospitals Coventry and Warwickshire NHS Trust, Coventry, UK; Canadian Cancer Trials Group, Queen’s University, Kingston, ON, Canada; MRC Clinical Trials Unit at UCL, Institute of Clinical Trials and Methodology, University College London, London, UK; MRC Clinical Trials Unit at UCL, Institute of Clinical Trials and Methodology, University College London, London, UK

## Abstract

**Background:**

Previous evidence supports androgen deprivation therapy (ADT) with primary radiotherapy as initial treatment for intermediate-risk and high-risk localised prostate cancer. However, the use and optimal duration of ADT with postoperative radiotherapy after radical prostatectomy remains uncertain.

**Methods:**

RADICALS-HD was a randomised controlled trial of ADT duration within the RADICALS protocol. Here, we report on the comparison of short-course versus long-course ADT. Key eligibility criteria were indication for radiotherapy after previous radical prostatectomy for prostate cancer, prostate-specific antigen less than 5 ng/mL, absence of metastatic disease, and written consent. Participants were randomly assigned (1:1) to add 6 months of ADT (short-course ADT) or 24 months of ADT (long-course ADT) to radiotherapy, using subcutaneous gonadotrophin-releasing hormone analogue (monthly in the short-course ADT group and 3-monthly in the long-course ADT group), daily oral bicalutamide monotherapy 150 mg, or monthly subcutaneous degarelix. Randomisation was done centrally through minimisation with a random element, stratified by Gleason score, positive margins, radiotherapy timing, planned radiotherapy schedule, and planned type of ADT, in a computerised system. The allocated treatment was not masked. The primary outcome measure was metastasis-free survival, defined as metastasis arising from prostate cancer or death from any cause. The comparison had more than 80% power with two-sided α of 5% to detect an absolute increase in 10-year metastasis-free survival from 75% to 81% (hazard ratio [HR] 0·72). Standard time-to-event analyses were used. Analyses followed intention-to-treat principle. The trial is registered with the ISRCTN registry, ISRCTN40814031, and ClinicalTrials.gov, NCT00541047.

**Findings:**

Between Jan 30, 2008, and July 7, 2015, 1523 patients (median age 65 years, IQR 60–69) were randomly assigned to receive short-course ADT (n=761) or long-course ADT (n=762) in addition to postoperative radiotherapy at 138 centres in Canada, Denmark, Ireland, and the UK. With a median follow-up of 8·9 years (7·0–10·0), 313 metastasis-free survival events were reported overall (174 in the short-course ADT group and 139 in the long-course ADT group; HR 0·773 [95% CI 0·612–0·975]; p=0·029). 10-year metastasis-free survival was 71·9% (95% CI 67·6–75·7) in the short-course ADT group and 78·1% (74·2–81·5) in the long-course ADT group. Toxicity of grade 3 or higher was reported for 105 (14%) of 753 participants in the short-course ADT group and 142 (19%) of 757 participants in the long-course ADT group (p=0·025), with no treatment-related deaths.

**Interpretation:**

Compared with adding 6 months of ADT, adding 24 months of ADT improved metastasis-free survival in people receiving postoperative radiotherapy. For individuals who can accept the additional duration of adverse effects, long-course ADT should be offered with postoperative radiotherapy.

**Funding:**

Cancer Research UK, UK Research and Innovation (formerly Medical Research Council), and Canadian Cancer Society.

## Introduction

When radiotherapy is used as initial treatment for clinically localised prostate cancer, it is often combined with androgen deprivation therapy (ADT). Multiple randomised controlled trials have compared different durations of ADT in patients having primary radical radiotherapy, who have not had previous radical prostatectomy. Improved long-term clinical outcomes have been observed with more extended durations of ADT, particularly in those with high-risk disease.^1^ Radiotherapy is also commonly used after radical prostatectomy, but only three phase 3 randomised controlled trials in this setting have assessed the addition of ADT, and none have compared different durations of ADT.

In people receiving salvage radiotherapy after radical prostatectomy, the addition of short-course (4–6 months) ADT has been shown in the RTOG 0534 randomised controlled trial to reduce disease progression^2^ and in the GETUG-AFU 16 randomised controlled trial to improve metastasis-free survival.^3^ The addition of long-course (24 months) bicalutamide to postoperative therapy in the RTOG 9601 trial improved not only metastasis-free survival, but also overall survival,^4^ at least in those with a higher prostate-specific antigen (PSA) level at the time of salvage treatment.^5^

In developing the RADICALS-HD trial in 2006, we hypothesised that long-course ADT would be more effective than short-course ADT in people receiving postoperative radiotherapy. We designed a prospective, international, randomised controlled trial to compare long-course versus short-course ADT in this setting. Given the results of a subgroup analysis of RTOG 9601 in 2020,^5^ we also wanted to assess any benefit from long-course versus short-course ADT with respect to comorbidity and PSA levels at the time of radiotherapy. The full background to the RADICALS trial, including the choice of outcome measures, is presented elsewhere.^6^

## Methods

### Study design and participants

RADICALS was an international, phase 3, multicentre, open-label, randomised controlled trial in prostate cancer. The protocol addressed questions regarding the timing of radiotherapy after surgery and the use of ADT with postoperative radiotherapy in separate randomisations with overlapping patient groups.

RADICALS-HD recruited patients due for radiotherapy at any time after previous radical prostatectomy for prostatic adenocarcinoma. The exclusion criteria were previous pelvic radiotherapy, preoperative ADT for longer than 8 months, any ADT within 6 months before surgery, PSA greater than 5 ng/mL, or metastatic disease, other active malignancy likely to interfere with protocol treatment or follow-up, or any postoperative hormone therapy. There were no age restrictions. Appropriate ethical review was in place for each participating country (appendix p 8). All participants gave written informed consent. The protocol is available online. This study is registered with the ISRCTN registry, ISRCTN40814031, and with ClinicalTrials.gov, NCT00541047.

### Randomisation and masking

Participants in the short-versus-long comparison of RADICALS-HD were randomly allocated to receive 6 months of ADT (short-course ADT) or 24 months of ADT (long-course ADT) in addition to radiotherapy. Site staff engaged patients about potential participation in the trial. Those who decided to participate were given the choice, with their clinical team, of being randomly assigned three-way 1:1:1 between no ADT, short-course ADT, and long-course ADT (adding 24 months of ADT) or two-way 1:1 either between just no ADT and short-course ADT or between just short-course ADT and long-course ADT. Sites were encouraged to randomly allocate patients three-way (none *vs* short *vs* long), but they could choose to allocate patients two-way (short *vs* long) if the patient was considered unsuitable for allocation to the no ADT group. Most participants in this comparison were allocated two-way. Participants allocated to no ADT are not included in this analysis. Randomisation was achieved centrally by the method of minimisation with a random element, stratified by Gleason score, positive margins, radiotherapy timing, planned radiotherapy schedule, and planned ADT type. The allocated treatment was open label.

### Procedures

ADT was to be initiated as soon as possible after randomisation, and certainly within 2 months. ADT was given with local choice of a subcutaneous gonadotrophin-releasing hormone analogue, supplemented by 3 weeks of an oral anti-androgen started 1 week before the first gonadotrophin-releasing hormone analogue administration. Monthly injections were recommended in the 6 months group and 3-monthly injections were encouraged in the 24 months group. Outside Canada, daily bicalutamide monotherapy 150 mg or monthly subcutaneous degarelix (with nationally approved dosing) were acceptable alternatives. Dose reductions were not possible; treatment could be stopped early if indicated. In participants for whom the end date of ADT was not recorded by sites, time on treatment summaries assume that ADT had not been continued indefinitely.

Radiotherapy was commenced approximately 2 months after starting hormone treatment. The intended radiotherapy schedule was prespecified for each participant as either 52·5 Gy in 20 fractions over 4 weeks or 66·0 Gy in 33 fractions over 6·5 weeks. The radiotherapy was to include the prostate bed and could also include pelvic lymph nodes. Detailed radiotherapy guidance was given in the protocol.

Scheduled follow-up was every 4 months for the first 2 years after randomisation, then every 6 months up to 5 years, and annually thereafter. PSA measurements were taken at every follow-up appointment and as clinically indicated. Imaging tests were done according to routine clinical practice and were reported locally, without masking of treatment allocation. There was no central review of imaging.

Clinician-reported data were collected at each follow-up visit on diarrhoea, proctitis, cystitis, haematuria, and urethral stricture, and were graded according to Radiation Therapy Oncology Group toxicity score.^7^ Data for other adverse events were collected if the event met the criteria to be classified as a serious adverse event.

The cause of death of trial participants was reviewed by a study clinician if there was uncertainty over whether the death was due to prostate cancer. An algorithm, without reference to allocated treatment, was used to identify deaths with uncertain cause, using the reported primary and contributory causes of death together with disease history during the trial. These participants with uncertain causes of death were centrally adjudicated by one of three clinical members of the Trial Management Group (CCP, NWC, or CNC).^8^ Additionally, for patients in England and Wales, national death registration data were available and included in the algorithm.

### Outcome measures

The primary outcome measure for RADICALS-HD was metastasis-free survival, defined as any distant metastasis or death from any cause. Secondary outcome measures were freedom from distant metastasis (any distant metastasis or death from prostate cancer), overall survival (death from any cause), initiation of non-protocol ADT, clinical progression-free survival (local or nodal progression, metastases, non-protocol ADT or death from prostate cancer), freedom from treatment failure (PSA progression when on ADT), toxicity, and patient-reported outcome measures (PROMs). An additional secondary outcome of treatment failure, defined as PSA progression when on ADT, was not well reported by sites and is not presented. PROMs were collected only in the subset of people also in the RADICALS-RT trial. This was a small subset so PROMs are not analysed here.

### Statistical analysis

This comparison was originally designed as part of a three-way comparison with disease-specific survival as the primary outcome measure and an overall recruitment target of 3053 patients across three arms. However, recruitment was permitted between pairs of arms to facilitate recruitment in an internal pilot, and accrual to these pairwise comparisons was more strongly supported by sites. In 2010, the trial was re-powered for separate comparisons of no ADT versus short-course ADT (reported elsewhere^9^) and short-course ADT versus long-course ADT. This was done without any reference to accumulating data within the trial. This separated, pairwise comparison required approximately 1077 patients to observe 91 events.

After recruitment and treatment had been completed for all participants, the primary outcome measure was subsequently brought forward to metastasis-free survival in 2019. This was done by the trial management group, who were not privy to accumulating comparative data in RADICALS-HD, following new evidence from the ICECaP study that metastasis-free survival was a robust early outcome measure for disease-specific survival. The full details of this change and the broader history of RADICALS are presented elsewhere.^6^ Based on 300 metastasis-free survival events from the 1523 participants, this revised design had 80% power with two-sided 5% α to detect an increase in 10-year metastasis-free survival from 75% to 81% (HR 0·72).

The full statistical analysis plan is published elsewhere^8^ and is summarised here. All analyses followed the intention-to-treat principle. Follow-up was estimated through reverse censoring on death. The statistical significance of differences between groups was evaluated with the log-rank test, stratified by randomisation minimisation factors. Effect estimates were obtained from Cox regression models, also stratified by randomisation minimisation factors. The Grambsch–Therneau test was used to test the proportional hazards assumption, with restricted mean survival time becoming the primary estimate of effect if non-proportional hazards were detected, with time restricted (t*) to 10 years. Time-to-event graphs were presented in KMunicate format^10^ Competing risk models were used for cause-specific survival with other causes of death as a competing risk. p<0·05 was considered to indicate statistical significance. Events rates as specified times were taken from Kaplan–Meier survival functions.

χ^2^ tests for heterogeneity or, where appropriate, trend were performed for consistency of effect. Two prespecified subgroup analyses were planned, by pre-radiotherapy PSA level and by Charlson Comorbidity Index score.^11^ We hypothesised that patients with higher pre-radiotherapy PSA, and those with less comorbidity, would benefit more from ADT. Exploratory subgroup analysis of all randomisation stratification factors was also planned. Forest plots were used to visualise the two pre-specified subgroup analyses. Multiple testing was taken into account when cautiously interpreting exploratory subgroup analyses.^8^ Safety was assessed in all randomly allocated participants.

The independent data monitoring committee (IDMC) met to review data from RADICALS on ten occasions. There were no formal stopping guidelines; the IDMC were asked to give advice on whether the accumulating data from the trial, together with results from other relevant trials, justified continuing recruitment of further patients or further follow-up. The IDMC did not recommend stopping the trial early.

### Role of the funding source

The funders of the study had no role in study design (other than organising initial peer review by independent reviewers), data collection, data analysis, data interpretation, or writing of the report. The sponsor took responsibility for these elements, delegated through their staff.

## Results

**Figure 1:**
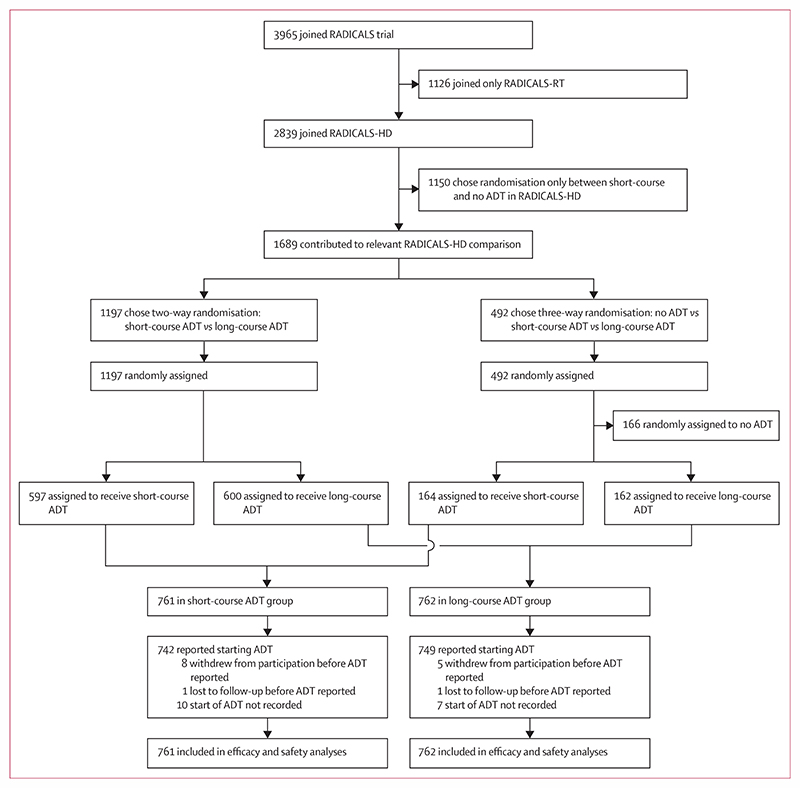
Trial profile ADT=androgen deprivation therapy.

Between Jan 30, 2008, and July 7, 2015, 1523 patients were randomly assigned to receive 6 months of ADT (short-course ADT group, n=761) or 24 months of ADT (long-course ADT group, n=762) in addition to postoperative radiotherapy at 138 trial-accredited centres in Canada, Denmark, Ireland, and the UK (figure 1). Of these 1523 participants, 1197 had been randomly allocated between only these two groups and 492 had been allocated to one of these groups as part of the RADICALS-HD three-way randomisation that also included no ADT.

The median age of participants was 65 years (IQR 60–69); 1407 (93%) had a Gleason score of 7 or higher and 461 (30%) had stage T3b disease or higher (table 1). Data on race and ethnicity were not collected. Radiotherapy was in the adjuvant setting for 653 (43%) patients and in the early salvage setting for 870 (57%) patients. The planned radiotherapy schedule was 66 Gy in 33 fractions for 1204 (79%) participants, and the radiotherapy target was the prostate bed alone for 1257 (85%).

Follow-up at sites for the trial ended on Dec 31, 2021: 1229 patients were still in follow-up at that date, 211 had died, and 83 had stopped their participation or become lost to follow-up. Median follow-up was 8·9 years (IQR 7·0–10·0). Among those still in active follow-up at the end of the trial, minimum follow-up was 5·1 years. The database was locked on May 27, 2022.

Median time from randomisation to starting hormone treatment was 6 days (IQR 0–14) in both groups. Median time to the last reported administration of ADT was 5 months (3–6) in the short-course ADT group and 21 months (6–23) in the long-course ADT group. 32 participants (19 in the short-course ADT group and 13 in the long-course ADT group) had no record on trial forms of starting treatment, 13 of whom formally withdrew from their participation in the trial (eight in the short-course ADT group and five in the long-course ADT group).

**Figure 2:**
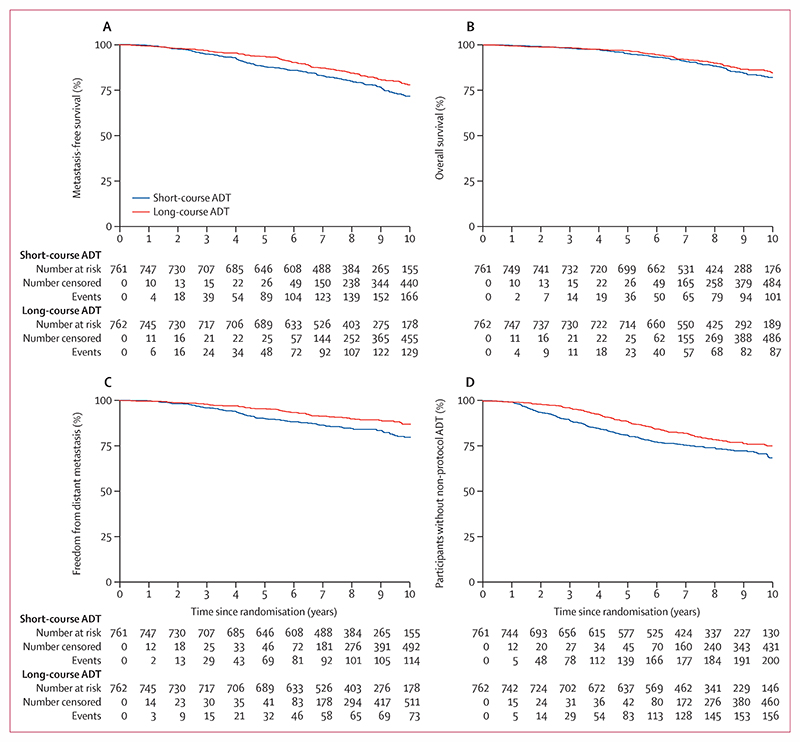
Primary and secondary outcome measures (A) Metastasis-free survival. (B) Overall survival. (C) Freedom from distant metastasis. (D) Time to non-protocol ADT. Risk tables present the number of participants who, at each timepoint, remain at risk, have been censored, or have had an event. All timepoints add up to the total number of patients. ADT=androgen deprivation therapy.

Metastasis-free survival events were reported for 313 patients, including 174 in the short-course ADT group and 139 in the long-course ADT group; 102 patients (63 short-course and 39 long-course) had metastases reported and were still alive at the end of the trial; 80 patients (49 short-course and 31 long-course) had metastases reported followed by death; and 131 (62 short-course and 69 long-course) died without having metastases reported. Metastasis-free survival was improved in patients allocated to long-course ADT compared with short-course ADT (HR 0·773 [95% CI 0·612–0·975]; p=0·029; table 2, figure 2). There was no evidence of non-proportional hazards. 10-year metastasis-free survival was 71·9% (95% CI 67·6–75·7) in the short-course ADT group and 78·1% (74·2–81·5) in the long-course ADT group.

**Figure 3:**
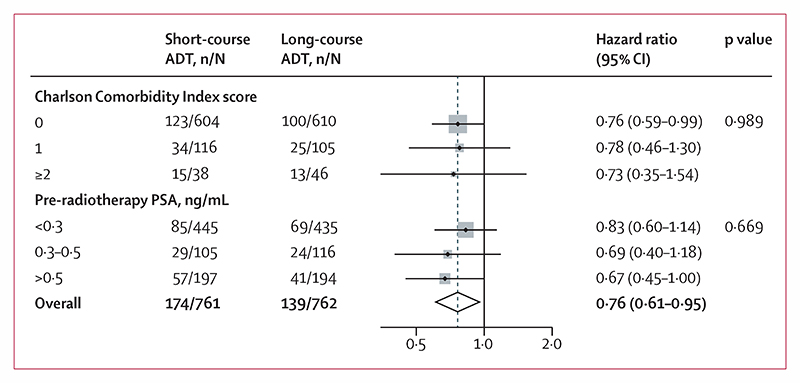
Pre-planned subgroup analyses Weighting is by sample size. ADT=androgen deprivation therapy. PSA=prostate-specific antigen.

The metastasis-free survival treatment effect did not differ meaningfully in either of the two prespecified subgroup analyses, pre-radiotherapy PSA level (interaction p=0·99) or Charlson Comorbidity Index score (interaction p=0·67; figure 3). There was also no evidence of differential treatment effect in the exploratory subgroup analyses by randomisation stratification factors; an interaction p value of 0·032 was observed with Gleason score but this was not considered statistically significant after allowance for multiple testing (appendix p 2).

Freedom from distant metastasis was improved in the long-course ADT group compared with the short-course ADT group (HR 0·634 [95% CI 0·471–0·853]; p=0·0024). In a competing-risks regression model with non-prostate cancer death as the competing risk, the sub-HR was 0·623 (0·467–0·831; p=0·0013). Time to clinical progression-free survival (0·728 [0·592–0·895]; p=0·0024) was improved in the long-course ADT group, although with clear evidence of non-proportional hazards; this is better summarised as improved restricted mean survival time from 8·12 years (95% CI 7·90–8·34) in the short-course ADT group to 8·73 years (8·55–8·91) in the long-course ADT group. Time to non-protocol ADT (0·733 [0·591 to 0·910]; p=0·0047) was improved in the long-course ADT group, although with clear evidence of non-proportional hazards; this is better summarised as improved restricted mean survival time from 8·32 years (8·11–8·53) in the short-course ADT group to 8·83 years (8·66–9·01) in the long-course ADT group. We found no evidence of a benefit to overall survival with long-course ADT. Causes of death are presented in the appendix (p 3).

During follow-up, Radiation Therapy Oncology Group scale toxicity of grade 3 or higher was reported for 105 (14%) of 753 participants in the short-course ADT group and 142 (19%) of 757 in the long-course ADT group (table 3). The most reported toxicities of grade 3 or higher were urethral stricture and haematuria. 24 serious adverse events were reported for 24 people in the short-course ADT group, including five reviewed as definitely or probably related to treatment. 49 serious adverse events were reported for 49 people in the long-course ADT group, including 13 reviewed as definitely or probably related to treatment. Three serious adverse events were reported fatal, none of which were reported as definitely or probably related to trial treatment (appendix p 4).

## Discussion

In this randomised controlled trial of patients receiving postoperative radiotherapy after previous radical prostatectomy, long-course ADT for 24 months was more effective than short-course ADT for 6 months in terms of metastasis-free survival, a clinically important long-term outcome measure. However, this benefit did not translate into an improvement in overall survival with a median of 9 years of follow-up. These results indicate that, on average, 16 people need to be treated with long-course ADT for one of them to avoid an metastasis-free survival event within 10 years. This metastasis-free survival benefit should be weighed against the extended duration of the well-known adverse effects associated with ADT, such as sexual dysfunction, metabolic syndrome, and osteoporosis. In addition, after 24 months of ADT, testosterone recovery is often prolonged or incomplete,^12^ so any adverse effects can be long-lasting.

These results are largely consistent with previous trials that had generated the hypothesis that long-course ADT would be more effective than short-course ADT. Of the two previous phase 3 trials of short-course ADT in the salvage radiotherapy setting, only one found a metastasis-free survival benefit, and neither showed any improvement in overall survival (HR 0·93 in GETUG-AFU 16 and HR 0·87 in RTOG 0534).^2,3^ The only trial of long-course hormone therapy, RTOG 9601 (which used bicalutamide monotherapy rather than ADT), reported an improvement in not just metastasis-free survival, but also in overall survival (HR 0·77).^4^ Taken together with RADICALS-HD, patients receiving postoperative radiotherapy seem to benefit more from the addition of long-course, rather than short course, ADT.

Patients and clinicians, when deciding on the duration of ADT to use with postoperative radiotherapy, will need to weigh up the benefits and harms of an extended duration of ADT. The harms of ADT are substantial, are well known, and matter to patients.^13^ In the current trial population, the relative benefit in metastasis-free survival translated into an absolute benefit at 10 years of 6%. Assuming a fixed relative benefit, the absolute benefit will vary according to baseline disease characteristics. It is therefore likely that patients with early PSA failure after radical prostatectomy, with a rapid PSA doubling time and high Gleason score, might experience a greater absolute metastasis-free survival benefit than those with more favourable characteristics. The clinical decision should also consider life expectancy. In the current trial population, the risk of death from prostate cancer was around 1% at 10 years. Therefore, any potential overall survival benefit from extended ADT is likely to be modest, particularly because the risk of death from other causes increases with age or comorbidity.

Subgroup analysis of RTOG 9601 found that the overall survival benefit from long-course bicalutamide was restricted to those with a higher PSA level at the time of salvage radiotherapy.^14^ By contrast, in the current trial, we found no evidence that the treatment effect differed according to PSA level. This apparent discrepancy might reflect, at least in part, differences in the distribution of PSA levels at the time of study entry, and hence differences in the PSA cutoffs chosen for subgroup analysis. Median PSA in RTOG 9601 was 0·7 ng/mL, which is substantially higher than the median PSA of 0·22 ng/mL in RADICALS-HD, a trial focused on early salvage radiotherapy. In RADICALS-HD, too few patients had pre-radiotherapy PSA of greater than 0·7 to test for the effect seen in RTOG 9601. There was no evidence of differential effects in any of the exploratory subgroup analyses (appendix p 2). Based on the RTOG 9601 results, bicalutamide is sometimes used in this setting, rather than a gonadotrophin-releasing hormone analogue. However, in RADICALS-HD, too few patients were treated with bicalutamide to draw any conclusions specifically about bicalutamide duration.

At present, there is no good way of predicting which people would benefit from long-course ADT. The DADSPORT meta-analysis (registered on PROSPERO, CRD42022325769) will include data from all four phase 3 randomised trials to date, incorporating the findings from other parts of RADICALS-HD,^9^ and might help to define which patients could benefit most from ADT. Genomic classifiers applied to the radical prostatectomy specimens might also help to predict benefit from salvage treatment. A study using the Decipher genomic classifier on 352 cases from RTOG 9601 generated the hypothesis that the survival benefit from 2 years of hormone therapy was less in individuals with a lower genomic classifier score.^14^ It would be of interest to test this hypothesis with samples from RADICALS-HD.

The strengths of RADICALS-HD include the large number of patients randomly allocated, international recruitment, and long-term follow-up. RADICALS-HD also has several limitations. Based on the recent results from the RADICALS-RT trial^15,16^ and the ARTISTIC meta-analysis,^7^ postoperative radiotherapy is now typically given in the salvage, rather than the adjuvant, setting. Around 43% of patients in this short-course versus long-course comparison in RADICALS-HD received radiotherapy in the adjuvant setting. However, there was no evidence of a differential effect of ADT duration according to the timing of radiotherapy. The majority of patients received radiotherapy to the prostate bed alone, although results from RTOG 0534 showed some support for radiotherapy to the pelvic nodes in addition to the prostate bed. It remains unclear whether the benefit of long-course ADT might differ in patients receiving pelvic nodal radiotherapy. Testosterone recovery measurements were not done in this pragmatic trial. Although the trial compared 6 months versus 24 months of treatment, patients receiving gonadotrophin-releasing hormone analogues will have experienced testosterone suppression beyond the treatment period. The trial opened around 15 years ago, and race and ethnicity data were not routinely recorded in UK-led trials at that time. Since data were not collected on ethnicity and race, we cannot comment on how well the participants reflect the underlying population, especially in light of well known differences in prevalence;^17^ the trial would not have been powered to look reliably for consistency of effect by ethnicity and race. This comparison recruited more than 1500 patients, but there were too few events to test any effect on overall or cancer-specific survival. Based on the evidence from ICECaP that metastasis-free survival can serve as a useful intermediate outcome measure, it remains plausible that long-course ADT will improve overall survival. This trial was in active follow-up during the COVID-19 pandemic from 2020 onwards. Recruitment had been completed many years previously so neither accrual nor allocation to short-course or long-course ADT would have been affected. There is no good reason to think follow-up would be impacted separately by allocated treatment group during the pandemic.

RADICALS-HD was done at a time when bone scan and CT scan were the conventional imaging techniques in use. More recently, prostate-specific membrane antigen (PSMA) PET has been introduced. PSMA-PET is more sensitive than the conventional techniques for the detection of metastatic disease and so would be expected to increase the metastasis-free survival event rate. However, if PSMA-PET had been used at the time of accrual to the trial, some patients with no evidence of metastatic disease on conventional imaging, but with metastases on PSMA-PET, would have been excluded. This would have had the opposite effect, lowering the metastasis-free survival event rate.

In summary, RADICALS-HD found that 24 months of ADT, in comparison with 6 months of ADT, improved metastasis-free survival in people receiving postoperative radiotherapy after radical prostatectomy for prostate cancer. This finding was consistent across all prespecified subgroups, including baseline PSA. For individuals who can accept the additional duration of adverse effects, long-course ADT should be offered in addition to postoperative radiotherapy.

## Supplementary Material

Supplementary material

## Figures and Tables

**Table 1:** Participant characteristics and pre-randomisation planned treatment

	Short-course ADT (n=761)	Long-course ADT (n=762)	All (n=1523)
Age, years	65 (60–69)	65 (61–69)	65 (60–69)
PSA at randomisation, ng/mL	0·22 (0·10–0·50)	0·24 (0·10–0·50)	0·23 (0·10–0·50)
Gleason score			
<7	61 (8%)	53 (7%)	114 (7%)
3 + 4	263 (35%)	267 (35%)	530 (35%)
4 + 3	220 (29%)	223 (29%)	443 (29%)
>7	215 (29%)	219 (29%)	434 (29%)
Missing	2	0	2
T stage			
1–2	206 (28%)	215 (28%)	421 (28%)
3a	327 (43%)	309 (41%)	636 (42%)
3b–c	215 (28%)	220 (29%)	435 (29%)
4	11 (1%)	15 (2%)	26 (2%)
Missing	2	3	5
Lymph node involvement			
Node negative	441 (58%)	429 (56%)	870 (57%)
Node positive	63 (8%)	66 (9%)	129 (8%)
No dissection	257 (34%)	267 (35%)	524 (34%)
Positive margins			
Absent	281 (37%)	278 (36%)	559 (37%)
Present	480 (63%)	484 (64%)	964 (63%)
CAPRA-S score			
Low (0–2)	57 (8%)	64 (8%)	121 (8%)
Intermediate (3–5)	315 (41%)	296 (39%)	611 (40%)
High (≥6)	388 (51%)	397 (52%)	785 (52%)
Missing	1	5	6
Country			
UK	515 (68%)	530 (70%)	1045 (69%)
Canada	210 (28%)	202 (27%)	412 (27%)
Denmark	35 (95%)	29 (4%)	64 (4%)
Ireland	1 (<1%)	1 (<1%)	2 (<1%)
Timing of radiotherapy			
Adjuvant	328 (43%)	325 (43%)	653 (43%)
Early salvage	433 (57%)	437 (57%)	870 (57%)
Planned RT schedule			
52·5 Gy in 20 fractions	145 (19%)	148 (19%)	293 (19%)
66·0 Gy in 33 fractions	604 (79%)	600 (79%)	1204 (79%)
Other	11 (1%)	13 (2%)	24 (2%)
Missing	1	1	2
Planned radiotherapy target			
Prostate bed	645 (85%)	642 (84%)	1287 (85%)
Prostate bed plus lymph nodes	115 (15%)	119 (16%)	234 (15%)
Missing	1	1	2
Planned hormone therapy			
LHRH agonist	640 (84%)	636 (84%)	1276 (84%)
Bicalutamide	119 (16%)	124 (16%)	243 (16%)
LHRH antagonist	1 (<1%)	0	1 (<1%)
Missing	1	2	3

Data are median (IQR), n (%), or n. Percentages were calculated using the number of participants with available data as the denominator. ADT=androgen deprivation therapy. PSA=prostate-specific antigen. CAPRA-S=Cancer of the Prostate Risk Assessment Post-Surgical. LHRH=luteinising hormone-releasing hormone.

**Table 2:** Primary and secondary outcome measures

	Short-course ADT (n=761)	Long-course ADT (n=743)	HR (95% CI)*	Log-rank p value*	Proportional hazards p value†
**Metastasis-free survival**					
Events	174	139	0·773 (0·612–0·975)	0·029	0·078
Metastases first	112	70	··	··	··
Prostate cancer death first	5	6	··	··	··
Death from other causes first	57	63	··	··	··
RMST (95% CI)‡	8·87 (8·70–9·03)	9·12 (8·97–9·27)	··	··	··
10-year metastasis-free survival (95% CI)	71·9% (67·6–75·7)	78·1% (74·2–81·5)	··	··	··
**Overall survival**					
Events	111	100	0·880 (0·663–1·169)	0·38	0·56
RMST (95% CI)‡	9·39 (9·27–9·51)	9·45 (9·34–9·57)	··	··	··
10-year overall survival (95% CI)	82·0% (78·3–85·2)	84·6% (81·0–87·5)	··	··	··
**Freedom from distant metastasis**					
Events	117	76	0·634 (0·471–0·853)	0·0024	0·15
RMST (95% CI)‡	9·16 (9·00–9·31)	9·47 (9·34–9·59)			
10-year freedom from distant metastasis (95% CI)	80·8% (77·0–84·1)	87·6% (84·4–90·2)	··	··	··
**Clinical progression-free survival**					
Events	222	173	0·728 (0·592–0·895)	0·0024	<0·0001
RMST (95% CI)‡	8·12 (7·90–8·34)	8·73 (8·55–8·91)	··	··	··
10-year clinical progression-free survival (95% CI)	66·5% (62·4–70·3)	73·1% (69·1–76·6)	··	··	··
**Time to non-protocol ADT**					
Events	200	157	0·733 (0·591–0·910)	0·0047	0·0001
RMST (95% CI)‡	8·32 (8·11–8·53)	8·83 (8·66–9·01)	··	··	··
10-year freedom from non-protocol ADT (95% CI)	68·5% (64·2–72·4)	75·1% (71·2–78·5)	··	··	··

ADT=androgen deprivation therapy. HR=hazard ratio. RMST=restricted mean survival time.

* Adjusted for randomisation stratification factors.

† Grambsch–Therneau test of non-proportional hazards.

‡ Restricted to 10 years.

**Table 3:** Maximum toxicity grade reported on Radiation Therapy Oncology Group scales

	Short-course ADT group (n=761)				Long-course ADT group (n=762)			p value^*^
	Grade 1–2	Grade 3	Grade 4		Grade 1–2	Grade 3	Grade 4	
Any toxicity	457 (60%)	99 (13%)	6 (1%)		449 (59%)	138 (18%)	4 (1%)	0·035
Diarrhoea	316 (42%)	10 (1%)	0		359 (47%)	13 (2%)	0	0·071
Proctitis	243 (32%)	16 (2%)	0		253 (33%)	25 (3%)	0	0·30
Cystitis	237 (31%)	13 (2%)	0		249 (33%)	22 (3%)	1 (<1%)	0·27
Haematuria	196 (26%)	39 (5%)	2 (<1%)		170 (22%)	51 (7%)	2 (<1%)	0·29
Urethral stricture	63 (8%)	53 (7%)	4 (1%)		76 (10%)	74 (10%)	1 (<1%)	0·070

Data are presented as n (%). Data were missing for eight participants in the short-course ADT group and five participants in the long-course ADT group. No grade 5 events were recorded. ADT=androgen deprivation therapy.

* χ^2^ test for trend.

## Data Availability

The RADICALS trial data are held at the MRC Clinical Trials Unit at University College London (UCL), which encourages optimal use of data by using a controlled access approach to data sharing. Requests for data can be made at any time and can be initiated by email to mrcctu. datareleaserequest@ucl.ac.uk or via our website. There is a formal application process, whereby the request will undergo review by the trial team, as well as independent researchers, to ensure that the proposed research is both ethical and has a strong scientific rationale. Data will not be released if this would compromise the ongoing research. The specific data and associated documents to be shared will be dependent on the nature of the individual request and this will be documented in a formal data sharing agreement.

## Research in context

### Evidence before this study

Three phase 3 randomised controlled trials have previously assessed the addition of androgen deprivation therapy (ADT) to postoperative radiotherapy after radical prostatectomy, and none have compared different durations of ADT. Short-course (4–6 months) ADT reduced disease progression and might improve metastasis-free survival (RTOG 0534 and GETUG-AFU 16) compared with no ADT. Long-course (24 months) bicalutamide improved not only metastasis-free survival, but also overall survival (RTOG 9601) compared with no ADT.

### Added value of this study

To our knowledge, this is the first trial to compare different durations of ADT with postoperative radiotherapy after radical prostatectomy in prostate cancer. ADT for 24 months was more effective than ADT for 6 months in terms of metastasis-free survival. This metastasis-free survival benefit should be weighed against the extended duration of the adverse effects associated with ADT.

### Implications of all the available evidence

For individuals who can accept the additional duration of adverse effects, long-course ADT should be offered with postoperative radiotherapy.
